# Clinical implementation, logistics and workflow guide for MRI image based interstitial HDR brachytherapy for gynecological cancers

**DOI:** 10.1002/acm2.12736

**Published:** 2019-10-10

**Authors:** Hualin Zhang, Eric D. Donnelly, Jonathan B. Strauss, Zhuang Kang, Mahesh Gopalakrishnan, Plato C. Lee, Gocha Khelashvili, Chithra K. Nair, Brian H. Lee, Vythialingam Sathiaseelan

**Affiliations:** ^1^ Department of Radiation Oncology Robert H. Lurie Comprehensive Cancer Center Northwestern University Feinberg School of Medicine Northwestern Memorial Hospital Chicago IL USA

**Keywords:** gynecological cancer, high dose rate, interstitial brachytherapy, MRI based brachytherapy

## Abstract

Interstitial brachytherapy (IBT) is often utilized to treat women with bulky endometrial or cervical cancers not amendable to intracavitary treatments. A modern trend in IBT is the utilization of magnetic resonance imaging (MRI) with a high dose rate (HDR) afterloader for conformal 3D image‐based treatments. The challenging part of this procedure is to properly complete many sequenced and co‐related physics preparations. We presented the physics preparations and clinical workflow required for implementing MRI‐based HDR IBT (MRI‐HDR‐IBT) of gynecologic cancer patients in a high‐volume brachytherapy center. The present document is designed to focus on the clinical steps required from a physicist’s standpoint. Those steps include: (a) testing IBT equipment with MRI scanner, (b) preparation of templates and catheters, (c) preparation of MRI line markers, (d) acquisition, importation and registration of MRI images, (e) development of treatment plans and (f) treatment evaluation and documentation. The checklists of imaging acquisition, registration and plan development are also presented. Based on the TG‐100 recommendations, a workflow chart, a fault tree analysis and an error‐solution table listing the speculated errors and solutions of each step are provided. Our workflow and practice indicated the MRI‐HDR‐IBT is achievable in most radiation oncology clinics if the following equipment is available: MRI scanner, CT (computed tomography) scanner, MRI/CT compatible templates and applicators, MRI line markers, HDR afterloader and a brachytherapy treatment planning system capable of utilizing MRI images. The OR/procedure room availability and anesthesiology support are also important. The techniques and approaches adopted from the GEC‐ESTRO (Groupe Européen de Curiethérapie ‐ European Society for Therapeutic Radiology and Oncology) recommendations and other publications are proven to be feasible. The MRI‐HDR‐IBT program can be developed over time and progressively validated through clinical experience, this document is expected to serve as a reference workflow guideline for implementing and performing the procedure.

## 1 INTRODUCTION

Cervical and endometrial cancer are the most common gynecologic cancer worldwide.^1^ Because of the anatomic advantage of being able to place catheters directly within the tumor, a high dose can be given locally while still limiting the dose to neighboring organs. Due to the global effort to provide improved access to radiation therapy and the importance of brachytherapy in the management of gynecologic malignancies in recent decades, there has been an increase in the number of high‐dose rate (HDR) gynecologic (GYN) brachytherapy programs world‐wide 2. Brachytherapy has become an integral treatment for locally advanced cervical cancer and other gynecologic malignancies.^3^ In cervical cancer management, the use of brachytherapy is found to have significantly associated with higher cause‐specific survival and overall survival and is therefore considered as a vital component of therapy.^4^ For bulky cervical or endometrial cancer, the interstitial brachytherapy (IBT) is often utilized to treat in situations that are not amendable to intracavitary treatments.

Although CT (computed tomography) images can be used for HDR IBT, magnetic resonance imaging (MRI) has been shown to be superior to CT in soft tissue delineation, which is helpful in accurate identification of the precise extent of the tumor.^5^, ^6^, ^7^ However, because most radiation oncology departments do not have an MRI scanner within the department, the proper handling of MRI images in brachytherapy planning can be challenging to both physicists and physicians. Considering the substantial technical barriers to implementing and commissioning the procedure, a document providing a practical and easy‐to‐follow logistics, workflow and guideline can be helpful for a beginner to start a new MRI based HDR IBT (MRI‐HDR‐IBT) program, and for an existing program to find the pros and cons of peer groups.

Aimed at accomplishing that goal, this document presents the categorized physics preparation steps, a sequenced workflow and verified implementation checklists following the AAPM medical physics practice guideline.^8^ A tabulated dosimetric metrics table was also presented. While the current document examines GYN IBT in detail, the basic techniques and preparation steps are also relevant for other anatomic sites. In this document we have chosen to focus our efforts on MRI‐HDR‐IBT of GYN origin, because the IBT of these cancer types has limited workflow guidelines in the literature compared to other types of brachytherapy procedures.^9^ The primary recommendations are based on GEC‐ESTRO publications,^10^, ^11^ but many of the specific techniques, checklists and work‐flow recommendations are derived from our institution’s experience.

Recently the TG‐100, an AAPM task group focused on the application of risk analysis methods to radiation therapy quality management, has published the recommendations for establishing radiotherapy quality assurance (QA) programs and clinical procedures with the cross verified process‐trees using a failure modes effects analysis (FMEA) or fault tree analysis (FTA).^12^ Since the MRI ‐HDR‐IBT involves many sub‐steps and procedures, this document has generated a workflow chart and FTA chart following the TG‐100 recommendations, and the potential errors and solutions as well.

## 2 METHODS AND MATERIALS

### 2.1 Preparation of MRI compatible equipment

#### 2.1.1 Interstitial catheters and MRI compatible line markers

Some radiation oncologists prefer to use metal catheters (i, e., needles) for IBT, because metal catheters are rigid and their insertion is felt to be easier than more flexible plastic catheters.^13^ However, the titanium catheters that were once classified as MRI compatible previously have been removed from the CT/MRI compatible equipment list by NIH owing to the distortions of surrounding tissue caused by the titanium needle artefact.^14^ It has also been reported that precise localization of the titanium needle tip is often difficult,^15^ and tumor delineation may be obscured by MRI signal cancellation of titanium material.^16^ As a result, our institution favors the use of plastic catheters with metal obturators to assist insertion. Plastic catheters are CT/MRI compatible; the hollow tubes of the catheters are dark and easily identifiable on CT imaging. However, plastic catheters are also dark on MRI T2 imaging and can be difficult to identify and track without the assistance of either CT fusion or the insertion of the MRI line markers. The MRI line markers are commonly thin plastic lines or thin plastic tubes filled with an MRI contrast enhancement agent. They are slightly thinner than treatment catheters, so they can be inserted into the catheters before MRI scanning to make the catheters more easily trackable for brachytherapy planning in MRI image.

**Figure 1:**
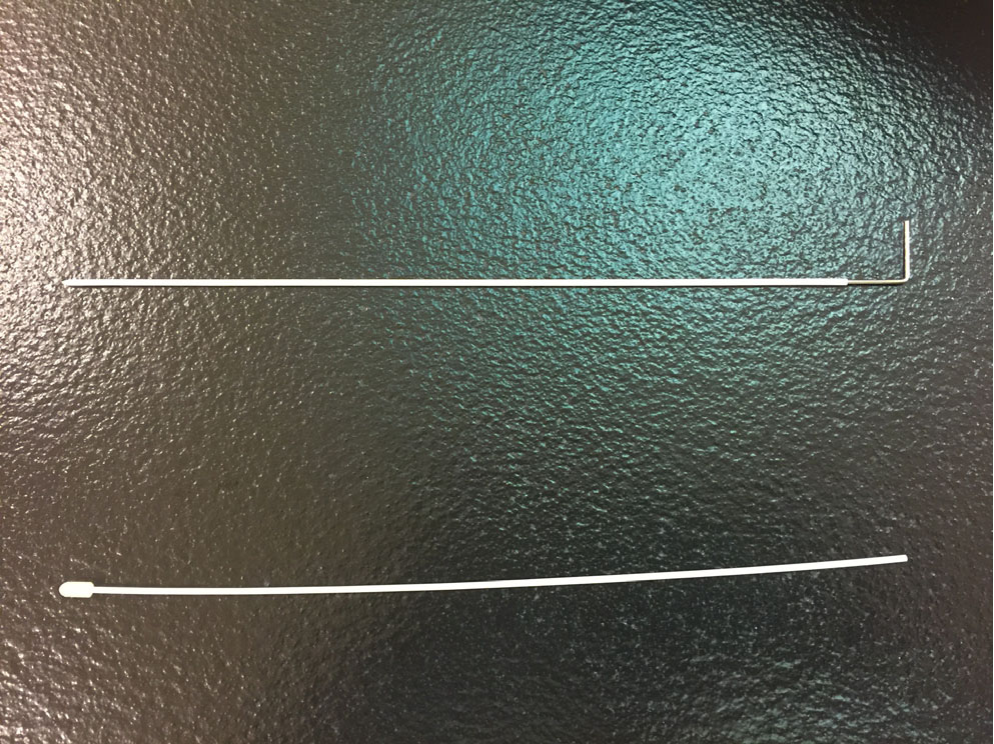
Two plastic interstitial catheters and a metal obturator. This picture shows an interstitial catheter with the metal obturator and an interstitial catheter with the protective cap and the obturator removed.

Catheters used at our institution are 18 G plastic catheters (Fig. 1) inserted with stainless steel obturators (Best Medical International, Inc, 7643 Fullerton Road, Springfield, Virginia) for insertion assistance. The catheter length is 274 mm. During the commissioning process five catheters from five different packages were measured, confirming that the uncertainty of length was within 2 mm. The consensus index length of catheters for Oncentra TPS (Oncentra, version 3.5, Elekta) and MicroSelectron^TM^ HDR afterloader (Nucletron, Elekta AB, Stockholm, Sweden) is determined by the Elekta source position simulator (SPS) and verified by experimental film tests (In our system, the consensus index length is 1251 mm. Reader needs to get their own). For each new batch of catheters, a physicist is required to verify the length from a randomly picked catheter, making sure the purchase order was accurately carried out and manufacturing uncertainty is within expectations (±2 mm).

The graphical offset of first dwell point relative to the catheter tip is an important parameter for calculating accurate dose distributions of the targets and organs at risk (OARs). By taping a catheter onto Gafchromic film (Ashland, Inc., Covington, KY), programming a dwell point at the most distal source position of the catheter (e.g., the consensus index length), and delivering radiation over a duration of 0.5 s, the graphical offset from the first physical dwell point to the catheter tip was determined to be 6 mm (±1 mm). Based on the average value of measurements, 6 mm is utilized as the consensus offset in CT‐based plan for our HDR afterloader. For the MRI‐based plan, the catheter has a different visual structure. The catheter tube itself has a 2−3 mm solid end at the tip, and the tip of the MRI line marker tube has a 2–3 mm solid end as well. These solid ends are not visible on MRI images when the catheters are tracked, thus the 6 mm offset seen from the film and CT is compensated by the solid ends in the MRI image, so the graphical offset of the catheter in the MRI‐based plan is approximated as 0.

#### 2.1.2 MRI dry‐runs and preparation of MRI line markers

Since the catheters are not identifiable on gynecological T2 images without the visualization assistance of MRI line makers, preparing MRI line markers is an important component of the procedure. It should be noted that, because MRI scanners vary in their characteristics, the image quality may be different between institutions and individual scanners, and therefore a verification by the brachytherapy physicist is required. Prior to the actual clinical use of MRI images for brachytherapy, we separately performed as many as 10 dry‐runs with T2 scans to test the MRI image quality for using different equipment in our 3 tesla MRI scanner (Verio, Siemens Healthcare, Erlangen, Germany), this is because some devices need multiple times of tests to find optimal working conditions of the MRI scanner and devices themselves. The tested equipment included CT/MRI compatible patient transportation board, CT/MRI compatible immobilization devices (such as clamp board, elastic net‐fashioned underwear, packing materials), Syed template plate, IBT catheters, and perhaps the most challenging and important device ‐the MRI line markers (filled with the T2 contrast agents for catheter visualization enhancement). The MRI line markers were tested for multiple times with MRI scanner to identify the best visualization agent. There are some T2 MRI markers that have been reported in the literature that utilized water^17^ and CuSo4 solution.^18^ We tested a list of T2 contrast agents such as the radiology lab T2 agents (copper oxide and gadolinium solutions), medical saline and two types of nylon fishing lines in a water tank. The gel might be a better medium than water for testing the devices used in MRI imaging, but our department did not have gel for testing, so we did not use it. Of note, some difficulties were encountered during tests/dry runs, the problems were progressively resolved. The T2 MRI contrast agents recommended by MRI researchers at our institution were found to provide good visualization of plastic catheters when the surrounding medium was water. However, when these same agents were used in real cases of gynecology cancer patients, it was found that the catheters could not be accurately visualized given the gynecological cancer lesions were dark in the T2 MRI images like the catheters visualized by the tested agents. We performed additional dry runs in which we hoped the catheters could give a white hyperintense appearance. Two types of nylon fishing lines and a plastic tube filled with medical saline were tested in three real cases. It was found the plastic tubes (at 1 mm in diameter) filled with medical saline provided the best visualization of the catheters on T2‐weighted MRI images.

**Figure 2:**
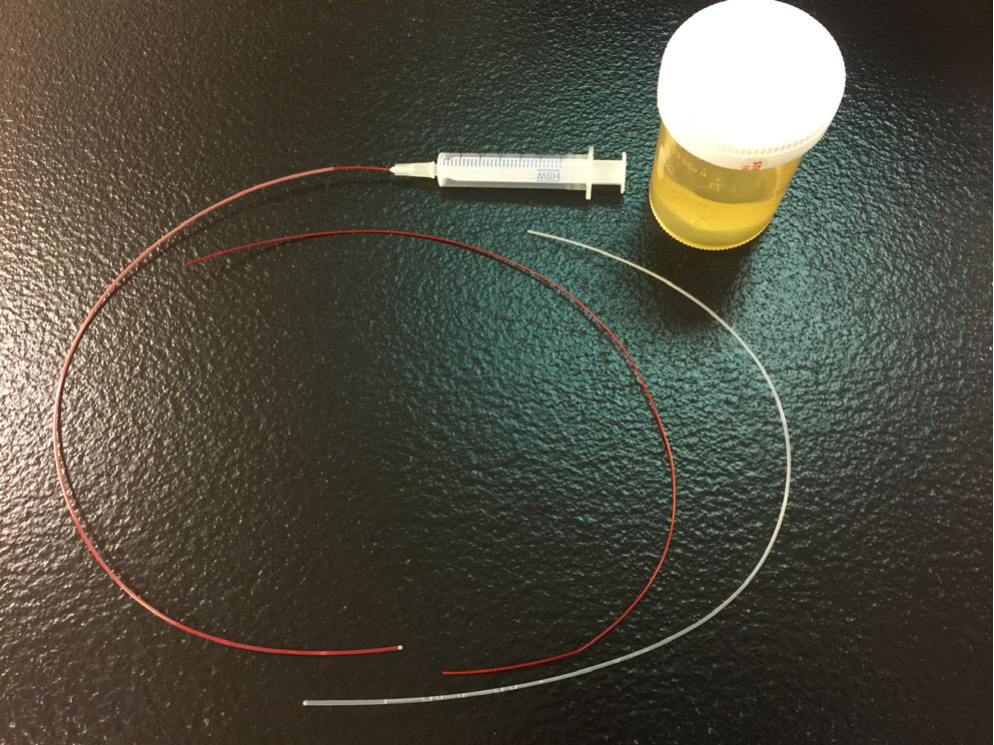
Equipment used to make the line markers of MRI‐HDR‐IBT MRI. Inside the plastic bottle is the prepared T2 contrast agent: medical saline plus several drops of iodine. The larger diameter plastic tubes are the line markers, and the smaller diameter red tubes are the tool used for filling saline into the line markers via the syringe.

Figure 2 shows the equipment used to prepare the MRI line markers for visualization of the IBT catheters on T2 images. The filling syringe needle is 30 G in diameter and 1/2 inch in length made by Becton and Dickinson Company (Hypodermic Catheters, PrecisionGlide^TM^, Product Number 305106. 1 Becton Drive, Franklin Lakes, NJ). The plastic line marker tube and the red thinner tube used to fill saline into the line marker tube are available through the Best Medical International Incorporation. Serval drops of iodine were added to the medical saline solution to enable visualization of potential air bubbles created in the process of saline filling. The air bubbles must be removed from the prepared MRI line markers because they will affect visualization quality.

#### 2.1.3 MRI compatible syed templates

**Figure 3:**
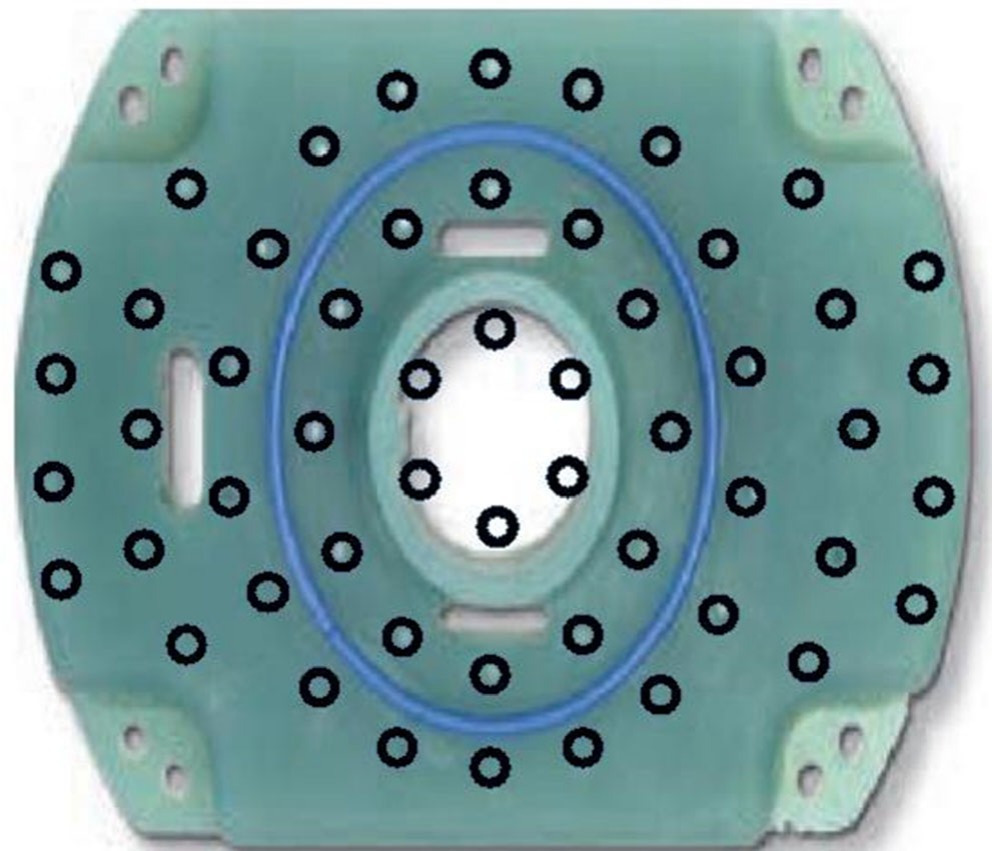
A Syed template provided by Best Medical International Inc.

There are a variety of MRI compatible templates available for IBT treatments. The template most commonly used at our institution is acquired through Best Medical International Incorporation and is refered to as the Syed template (Fig. 3), although they have been modified from the original design by Syed and Neblett.^19^ This tempalte has 54 insertion points, enabling a variety of loading patterns to treat diverse clinical situations not amenable to standard intracavitary approaches. The template is composed of plastic MRI/CT compatible material. The template itself can be clearly seen in CT image, but can be more difficult to differentiate from tissue on T2 MRI images. However, visualization of the catheters and catheter locations can be accomplished through insertion of the MRI line markers within the catheters.

### 2.2 MRI image acquisition and processing

#### 2.2.1 MRI image sequence selection and acquisition

Several studies have evaluated the best MRI sequences for gynecological brachytherapy.^10^, ^20^, ^21^, ^22^, ^23^ The MRI sequences used for our brachytherapy procedure follow the GEC‐ESTRO recommendations^22^ with some modifications based on input of diagnostic radiologists at our institution (see below for all sequences). In our institution, two 8‐channel phased torso coils provided by Siemens are used for all scans.

The MRI sequences acquired at our institution are:

- Scout
- Coronal haste (Coronal single shot T2),
- Axial Haste (Axial single shot T2)
- Sagittal haste (Sagittal single shot T2),
- AX 3D T2 TSE (3D Axial Turbo Spin Echo T2),
- Sagittal Vibe (Sagittal T1),
- Axial Vibe (Axial T1)

MRI scan is then paused while injecting T1 lesion contrast agent prior to proceeding to the following sequences:

- Sagittal Vibe 45 s (45 s delayed T1)
- Sagittal Vibe 90 s (90 s delayed T1)
- Axial Vibe (Axial T1).

The patient will stay on the MRI couch for about 45 min to complete all above 10 sequences. The sequence of AX 3D T2 TSE is the primary scan utilized for brachytherapy planning, the scan slice size is 1 mm for a better spatial resolution, and it takes 6–9 min. Other listed sequences are undertaken for additional assistance in tumor verification and radiologic interpretation, readers may not need them.

A successful treatment plan relies on the quality of MRI images. Given this, a rule was set at our institution that a medical physicist who is familiar with the MRI imaging protocol be physically present during MRI image acquisition.

*MR imaging checklist:*

**Figure 4:**
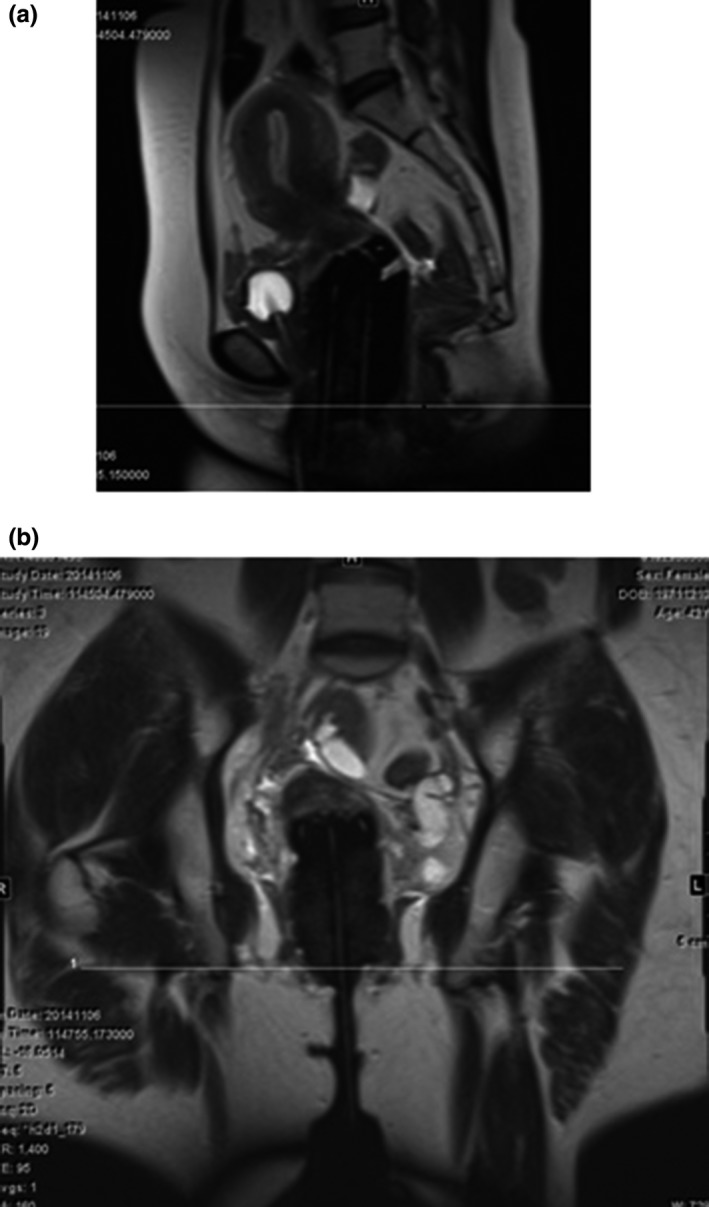
MRI sagittal view (a) and coronal view (b). Make sure to have sufficient margin (5 cm) above the uterus. MRI, magnetic resonance imaging.

**Figure 5:**
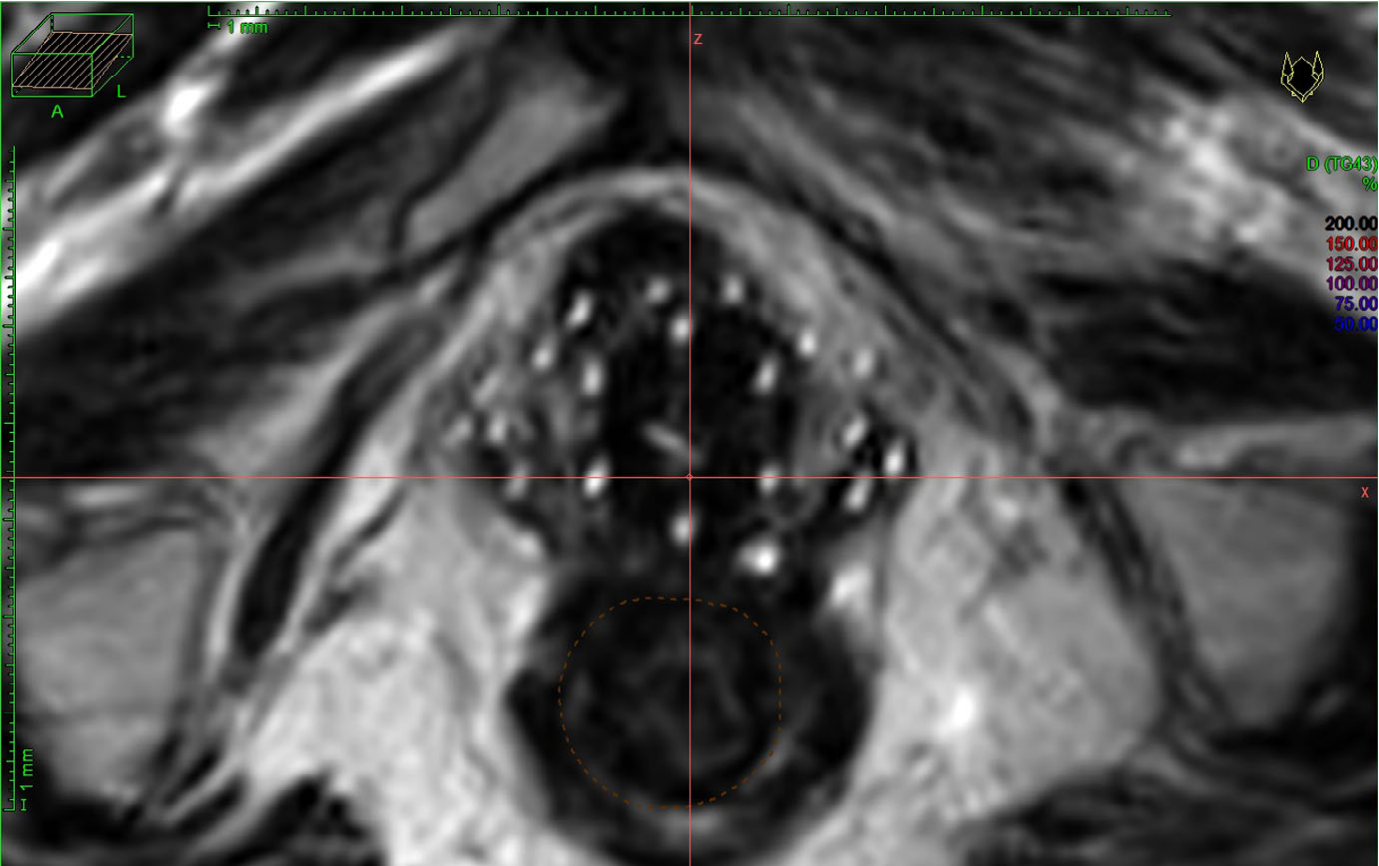
Catheters visualized by MRI line markers in T2 weighted MRI axial scan image. MRI, magnetic resonance imaging.

1. Assure that the patient is transported between the cart and the MRI scanner couch in a fashion that minimizes catheter disruption to avoid altering patient position or moving the implant. ____
2. Check that the MRI line markers are fully inserted into the catheters before scanning. ___
3. Verify that the patient is scanned head‐first since this is the default setting for most radiation oncology treatment planning systems (TPS). ___
4. Confirm the MRI sequences are from the gynecological brachytherapy MRI imaging protocol. ___
5. Check that the MRI scanning range is large enough to cover the whole pelvis (Fig. 4). The upper field border should extend 5 cm cephalad to the uterus. The lower field boarder should include the template plate. ___
6. Assure that the patient images are collected at a clear axial orientation, this is especially important for the T2 weighted image set used for treatment planning. ___
7. Make sure the line markers are visible in T2 image (see Fig. 5). ___
8. Make sure all slices of the axial T2 images were sent to brachytherapy TPS. ___

In the event the catheters are not visualized on the MRI T2 images, a simulation CT scan can be performed and fused with the MRI image set. An anterior‐posterior and a lateral scout images are acquired to check patient position on the CT couch first. Ideally, the patient will be centered in the bore to optimize and narrow the field of view of the subsequent images. A pelvic helical CT scan is performed using the following scanning parameters: 2‐mm slice thickness, contiguous increase, pelvis protocol.

#### 2.2.2 Handling MRI images in brachytherapy TPS

After MRI images are acquired, all series are transferred to the HDR computer. The axial and fast spin echo T2‐weighted MRI images (Fig. 5) will be used for planning. Most brachytherapy TPSs have a fixed protocol for handling multiple image modalities in a single plan. The most important step is the primary image must be loaded into the TPS first. The primary image is defined as the image to be used to track applicators or catheters for making the treatment plan. If the catheters can be tracked in the scanned T2 MRI image, then the T2 MRI image will be used as the primary image, the reconstruction of catheters, delineations of target volumes and OARs and development of treatment plans will be completed within it, the CT scan will not be needed.

In the case when the CT image had to be used as the primary image, the MRI image would become secondary image. All the OARs will be contoured from the CT image in addition to tracking all catheters. The target volumes will be delineated from the co‐registered T2 MRI image and then copied to CT image. The target volumes may have to be modified in the CT image to accommodate the registration uncertainty. Our test indicated that a violation of primary image rule will result in unacceptable catheter position determination errors and propagated dosimetric uncertainty within the treatment plan.

The treatment plan will be manually optimized using a graphical optimization method. Care is taken to assure adequate coverage of the target, sparing of the OARs, and avoidance of especially long dwell times in any one dwell position. At our institution patients most commonly have five fractions of 5–6 Gy per fraction delivered two fractions a day and separated by at least 6 h. Typically, only one treatment is performed on the afternoon of the first day given the time required for insertion, imaging and planning.

Our institution has treated over 30 patients with MRI‐HDR‐IBT, the MRI images were exclusively used for almost all but two cases for planning. In these two cases (happened in separate calendar year), the MRI line markers were failed to be visualized and CT scans had to be ordered. A later review of MRI image quality with an MRI technician revealed the MRI scanner might be in a poor performing condition on those days.

*The CT/MRI registration checklist:*

1. Make sure both the CT and MRI series are correct. ___
2. Make sure the CT image will be imported first, and the MRI image will be imported afterwards in the same case. ___
3. Select CT and T2 MRI images for registration. ___
4. Select: *Landmark* as the registration method. Use catheters for registration, use bony marks for assistance. ___
5. Make sure to use at least three pairs of points from CT and MRI images for registration. The registration points from the same modality (CT or MRI) should be located onto different slices. ___
6. Make sure the TPS reported registration error be less than 5 mm. ___
7. Make sure the registration result is approved by MD. ___
8. Make sure the target volumes are delineated from the MRI image and copied to CT images. ___
9. Make sure the OARs are delineated from the CT image. ___
10. All delineated structures were reviewed and approved by MD. ___

### 2.3 Target volumes and OARs

Although different institutions may decide to contour different target volumes and OARs, GEC‐ESTRO recommends specific structures for brachytherapy planning.^10^ Those structures are: a) GTV (gross target volume), b) HR‐CTV (high risk clinical target volume), c) IR‐CTV (intermediate risk clinical target volume), d) Bladder, e) Rectum, f) Sigmoid, g) Bowel‐bag.

GEC‐ESTRO recommendations describe the creation of the IR‐CTV by expanding the HR‐CTV with a margin of 1 cm excluding the OARs.^10^, ^11^

If the physician is interested in the doses of any additional points, the physicists can use “Patient Points” to mark these points of interest. The commonly used points of interest are the vaginal wall, the femoral heads, and the ICRU points of rectum and bladder points.^24^

Of note, many aspects of the patient’s care during the procedure are performed by physicians within other specialties. One of the most important such tasks performed is pain management. A thorough discussion with anesthesia team prior to the implementation of an MRI‐HDR‐IBT program is recommended. Additionally, patients are often admitted to the hospital and covered by an inpatient service such as gynecologic oncology. Importantly, prophylactic anticoagulation should be strongly considered given the combination of the risk factors of active malignancy and patient stasis. At our institution, Lovenox is commonly given daily to most patients during the course of IBT.

## 3 RESULTS

### 3.1 General strategies of planning

The optional planning strategies developed at our institution include:

- The case label should be written with the modality and fraction number, such as MRI plan Fx1, or CT plan Fx2. Thus, it can be clearly identified in the treatment control system (TCS) to avoid loading the wrong plan. This is particularly helpful if a modification of plan using the same set of images is necessary during the current IBT course. For example, when a catheter obstruction is discovered after first fraction of treatment, a new plan will be made based on the available catheters with the same images, the fraction number (such as MR plan Fx2) will help identify the new plan in the treatment consol.
- The plan property should list the applicator type, number of catheters, prescription dose and evaluation method. For example: Syed template, 15 catheters, 5 Gy. Plan optimization method: HR‐CTV coverage.
- Use a planning technique that you are familiar with. There are a variety of planning techniques available in modern brachytherapy TPSs, those techniques could be either manual or automatic IPSA (Inverse Planning by Simulated Annealing). A common practice is to turn off the dwell points which are located outside of the HR‐CTV, unless a part of the IR‐CTV volume needs significant attention. However, if no catheter or not enough catheters are inside portions of the HR‐CTV due to difficulty of catheter insertion, then you may need to turn on the dwelling points of catheters inside the IR‐CTV or close to the HR‐CTV. All dwell points will possess the same weighting factor 1.0.
- Create a list of points onto the surface of the HR‐CTV, then allow the TPS to normalize the plan with those points to make an initial basic plan. Starting from the initial basic plan, the MD will use the graphical optimization method to tune the plan for a desired coverage.

It should be noted that although the modern TPSs provide many convenient methods for manipulating dose distributions, matching the dose distribution to the HR‐CTV while simultaneously reducing the doses of the OARs can be challenging and time consuming. Achieving a proper dose distribution with HDR brachytherapy requires both accurate insertion of catheters and a careful optimization process.

### 3.2 Recommended planning and treatment workflow

In order for readers to easily manage their own workflow of planning and treating the patient, a planning checklist adopted at our institution is listed here for reference.

*MRI‐HDR‐IBT planning checklist:*

**Figure 6:**
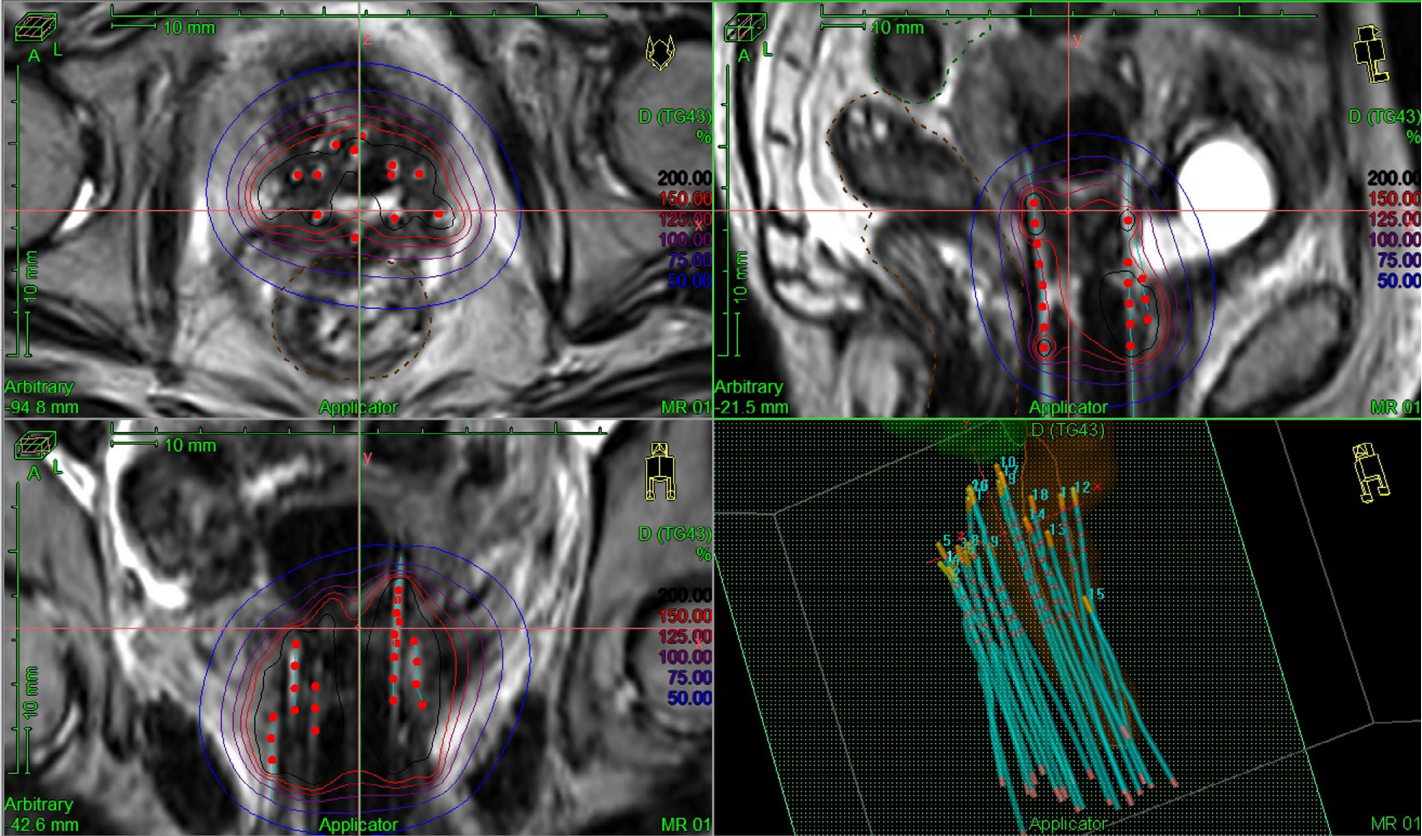
A typical case of MRI‐HDR‐IBT plan seen in a TPS. TPS, treatment planning systems.

1. Make sure the primary image (which is visible for MR line markers or visible for catheters if CT must be used) is loaded in TPS planning workspace. ___
2. Make sure the primary image is used for tracking the catheter. ___
3. Track catheters always starting from an axial slice at the inferior end of Syed template. Because at the inferior side or template slice, the catheters still keep the designed pattern, so you can easily identify and label all catheters. Conversely, the catheters located in the superior region may have been bent during insertion and the catheter locations may be difficult to ascertain. So, by going to a proper inferior slice on the axial view, the whole catheter pattern will be shown. (Fig. 5). ___
4. Start and set at: “Connector End” if your catheters are better identified from connect end. Otherwise you need to start tacking from “Tip End” if you want to start tracking from the tip of catheters. Check “Project Current Catheter” if this function is available. ___
5. Track catheters starting from the selected inferior slice to the superior slice until reaching the catheter tip, if the “Connector End” is used. It was found the work will be more efficient if the catheters are tracked in the manner of one catheter each time, since the catheters will likely reach different depths. ___
6. Check all tracked catheters from the 3D view, make sure they are reasonable. (Fig. 6) ___
7. Set the index lengths for the peripheral catheters and central tandem. ___
8. Set the graphical offsets. If the MRI is used as the primary image, the offset is 0 for all channels. If the CT image is used as the primary image, the offset of all catheters is −6 mm, of the tandem is −7 mm. ___
9. Mark ICRU Bladder and Rectum points and other points of interest in the patient coordinates system if they are requested by MD. ___
10. Enter the prescription dose and fraction number. ___
11. Map the channels. All catheter channels will be mapped based on the MD’s Syed template implant pattern. The tandem will be mapped to a specified channel. ___
12. Activate the dwell points inside the HR‐CTV. ___
13. Make a preliminary plan by normalizing dose to a list of reference points onto the surface of the HR‐CTV or use IPSA. ___
14. MD adjusted the plan. The final plan will achieve an adequate coverage to the HR‐CTV and the OAR doses as low as possible. ___
15. MD finalized and approved the plan. ___
16. Fraction dose and channel mapping were verified by MD ___
17. Target coverage was verified ____
18. The plan was checked by a second physicist. ___
19. Plan was exported to TCS. ___
20. Physicist printed the plan. MD and physicist signed the plan. ___
21. Plan was successfully executed. ___
22. Patient survey form was completed. ___

### 3.3 Treatment documentation

For a brachytherapy plan or course, the plan evaluation and dosimetric documentation are very important, because it will indicate if the delivered dose can achieve an expected tumor control (through the summed dose to the target volume) and if the OARs will exceed the expected toxicity level (through the OAR dosimetric metrics).

The following dosimetric documents are implemented in our institution:

- At the first fraction, MD will review and approve the dosimetric metrics (the DVH tables and iso‐coverage graphs),
- A physics consult document will be created at the first fraction and signed by MDs, The physics consult document will reflect the techniques used, image modalities utilized and physics equipment involved.
- If one or more channel(s) could not go through the obstruction test and had to be skipped during the treatments, an updated plan and DVH table reflecting the actual dose coverage must be provided. MD will review and sign the new DVH tables.
- For every following fraction, the obstruction check may find more undeliverable catheters. If some catheters cannot go through obstruction checks, as occasionally happened the dose coverage would be significantly deteriorated if the compromised plan was used. Under such a circumstance, a new plan may need to be created by the physics/physician team, thus the new plan will still be able to provide an adequate dose coverage after adjusting the dwelling times from the deliverable catheters. The new plan will be signed and approved by the MD, the new dosimetric metrics, the fraction number, the channel numbers and catheter labels used by the new plan will be documented.
- After the last treatment, all doses to the targets and OARs from all fractions will be summed, the physicist will provide the dose summary document. Table 1 lists the dosimetric metrics reported in the MRI‐HDR‐IBT summary document.

**Table 1:** Dosimetric metrics used in evaluation of MRI‐HDR‐IBT for gynecological cancer. The table shows the dosimetric metrics from a real case with 15 catheters. Data are from one‐fraction of treatment, the prescription dose is 5.5 Gy.

	Targets		
	GTV	HR‐CTV	IR‐CTV
D 100 = MTD D 100_iso_ [α/β = 10 Gy] (Gy)	3.5	3.8	1.8
D 90 D 90 _iso_ [α/β = 10 Gy] (Gy)	5.5	5.9	2.5
V 100 volume of PD [%]	100	93.6	20.5
V 90 Volume of 90% PD [%]	100	97.6	27.2

	OARs			
	Bladder	Rectum	Sigmoid	Bowel‐bag
D0.1 cm^3^ – dose (Gy) 0.1 cm^3^ – D_iso_ [α/β = 3Gy]	6.1	2.5	9.6	1.8
D1 cm^3^ – dose (Gy) 1 cm^3^ – D_iso_ [α/β = 3Gy]	4.1	1.6	6.0	1.5
D2 cm^3^ – dose (Gy) 2 cm^3^ – D_iso_ [α/β = 3Gy]	3.5	1.3	4.9	1.4

Abbreviations: OARs, organs at risk.

*Note:* D100 and D90 are respectively for the doses covering 100% and 90% of the target volumes. V100 and V90 are the percentages of target volumes covered by 100% and 90% of the prescription dose. D0.1 cm^3^. D1 cm^3^ and D2 cm^3^ are the maximum doses respectively received by 0.1 cm^3^, 1 cm^3^ and 2 cm^3^ of the OARs.

## 4 DISCUSSION

### 4.1 TG 100 analysis

**Figure 7:**
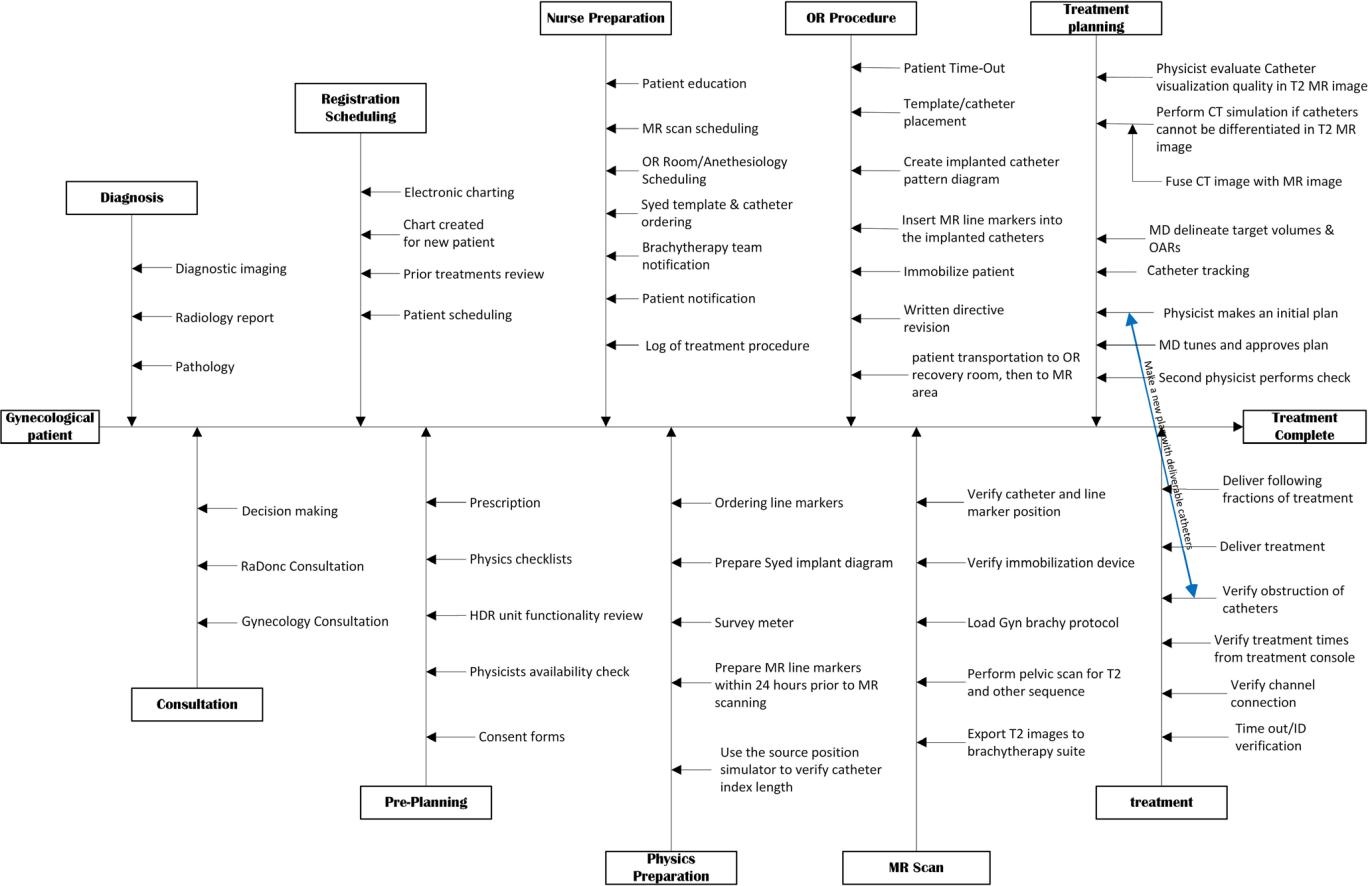
An example workflow for MRI‐HDR‐IBT following the AAPM TG‐100 guidelines.^12^

As we have seen, the complexity of the MRI‐HDR‐IBT is far beyond some intracavitary brachytherapy procedures such as the vaginal cuff or ring and tandem HDR brachytherapy. Therefore, both the work flow chart and FTA based on the TG‐100^12^ will help users to better understand and manage the procedure. In general, through multi‐disciplinary collaboration, the authorized medical physicist (AMP) is responsible for establishing quality standards and workflow for the MRI‐HDR‐IBT. The established workflow shall be fully understood by all team members. An example of MRI‐HDR‐IBT workflow is shown in Fig. 7.

It is noted that the workflow details will vary across institutions depending upon available resources and their unique situations.

Per TG‐100, after the workflow is well understood by the involved personnel, failure mode and effect analysis (FMEA) should be performed by the team to identify which processes cause high risk among the possible failure modes.^12^

**Figure 8:**
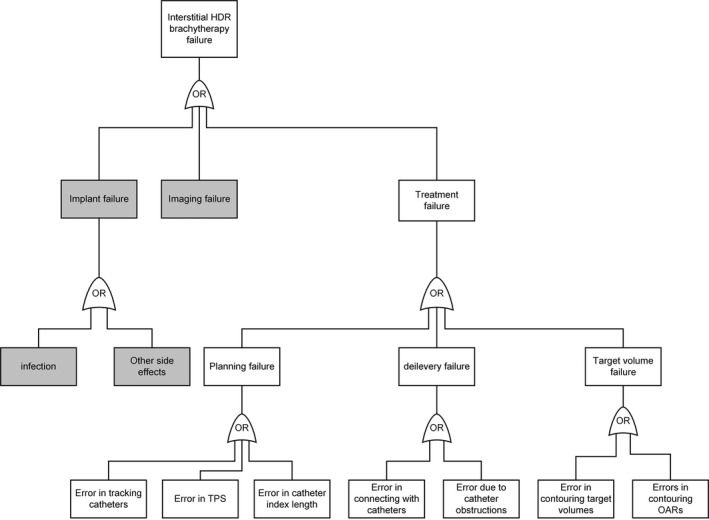
An example of MR guided interstitial HDR brachytherapy fault tree analysis. HDR, high dose rate.

Figure 8 is a FTA prepared for an MRI‐HDR‐IBT case. The fault tree began with the potential failure modes that would produce a failed/abandoned treatment due to imaging failure, or catheter implantation failure or treatment failure. The failures indicated in the grey boxes are solely of the physician/MR technician and are not considered further in this discussion.

As seen in Fig. 8, the causes for each potential failure mode were investigated and further brought to the downstream causes. Actions to address the potential failure modes can be to either eliminate the causes that can start propagation along the branch of the fault tree or to interrupt the failure progression by placing an intervention along the branch. Either method can be effective.

However, it should be remembered that the potential errors that we speculated may not be reflective at other institutions, there could be other errors which will equally cause the treatment failure in a particular institution or practice. The TG‐100 pointed out “there is little hard observational data available for populating FMEAs or FTAs with occurrence and detection probabilities. Most radiotherapy risk analyses are prospective models of planning and delivery based on the experience, expert knowledge, and expectations of the treatment team members who participate in the analyses. Medical error taxonomies, of which two have been developed specifically for radiation therapy,^25^, ^26^ are intended primarily to support root cause analysis.” Therefore, the error and solution table (Table 2) which was made based on our experience and expectations, is more for the readers’ reverence, the specifics on some of these errors plus the solutions to the errors pertinent to performing MRI‐HDR‐IBT are elaborated upon below mainly to aid in institutional discussions.

**Table 2:** Sub‐steps, errors, estimated impact factors and solutions.

Sub‐steps	Possible errors	Impact to treatment	Impact factor	Solutions to the errors
Line marker preparation	Bubbles were not removed; empty line markers were accidently handed to MD	Catheters will become not trackable in MRI image; CT image must be used.	8	Make line markers within 24 h of implant, check remove bubbles before use
MD catheter insertion	Catheters kinked; catheters converged to one location	If the most catheters are kinked or away from target, DVH coverage will be compromised.	10	Pre‐insertion MD training, a sketched plan for needle insertion location
MR scanning	Wrong protocol used. T2 imaging in poor working condition, MR line markers were not fully inserted. Catheter movements during rough movement/transportation.	Target delineation could be inaccurate, catheters cannot be tracked in MR image. Catheters are outside of target.	10	A physicist will verify the line markers and MR protocol before scan.
CT Scanning (If MR line markers were not visible in T2 MR image)	Forgot to pull MR line markers, rough movement/transportation displaced the catheters locations, wrong CT protocol, and CT/MR registration error.	Catheter tracking difficulty, image registration error, target and OARs volume delineation error.	10	A physicist will monitor the patient transportation and pull off line markers before scan.
Planning	Wrong volumes, wrong targets, inaccurate catheter tracking, channel mapping error, index length error, graphic offset errors, optimization error.	Patient could get inadequate treatment; treatment could not be executed.	10	MD will be familiar with GEC‐ESTRO protocol. A physics check list will be prepared for planning and second check. A sketch of needle locations and channel mapping plan will be confirmed by both the MD and physicist.
Treatment	Connect HDR channels with wrong catheters, catheters fell out,	Wrong dose delivered, mistreatment.	10	Physicist verify connected channels by comparing with the insertion diagram. The catheters to be used are marked at that depth + margin+ template thickness. MD documents catheter depths with a ruler at the fraction 1 and verify in following fractions.
Documentation	Dosimetric metrics calculation was inaccurate, biologically equivalent dose calculation was inaccurate, summation of BT and EBRT was not accurate.	Treatment outcome could be mistakenly explained, follow‐up treatment could be erroneously formulated.	5	The algorithm of dose calculation is verified by a second physicist, the equation is locked. The date entered into the spreadsheet are checked by 2nd physicist each time.

### 4.2 OR procedure and workflow

This document primarily focuses on the physics aspects of MRI‐HDR‐IBT, the OR procedure performed by MDs and nurses is briefly presented here just for helping physicists know what happened in the OR room. The interstitial catheter insertion is generally performed in the OR under general anesthesia. A Foley catheter is placed in the bladder. A vaginal obturator is placed through the Syed template center and is inserted into the patient’s vagina while in the dorsal lithotomy position ensuring the vaginal obturator is flush against the top of the vagina and the template is flush against the perineum. Once properly placed, the template is sutured in place to the perineum. After the template is securely in position, catheters are placed within the template. Pre‐operative imaging is utilized to determine extent of catheters, approximate placement for adequate coverage and depth required. Ultrasound imaging can be used to aid in determining the depth of insertion of catheters and coverage. In some clinical scenarios, especially when the uterus is surgically absent and small bowel may be adherent to the tumor or vaginal apex, laprascopic assistance may be helpful to avoid visceral puncture. Since the patient often remains immobile and supine for an extended duration, we favor a well‐cushioned cart and an air‐assisted patient transfer mat (Hovermatt, HoverTech International, Bethlehem, PA) underneath for assistance in gentle transportation. Patients receive pain medication while the instrumentation is in place most commonly through a patient‐controlled anesthesia (PCA) pump. After the devices are placed and recovering, the patient is brought to the radiology department. In order to minimize patient’s catheter movement, the MRI couch is moved to the patient’s holding area within the radiology department and the patient is directly transported onto the MRI couch, instead of using a third table for transitional transportation, this approach would further mitigate unnecessary patient’s catheter movement. After the MRI scans are complete, the patient is transported to the radiation oncology department holding area while the physicists and radiation oncologists create the treatment plan based on the MRI images.

An implant diagram reflecting the locations and numbers of catheters inserted during the OR procedure will be drawn by MD on a schematic Syed template graphic as a reference.

### 4.3 Times spent in different sub‐procedures

**Figure 9:**
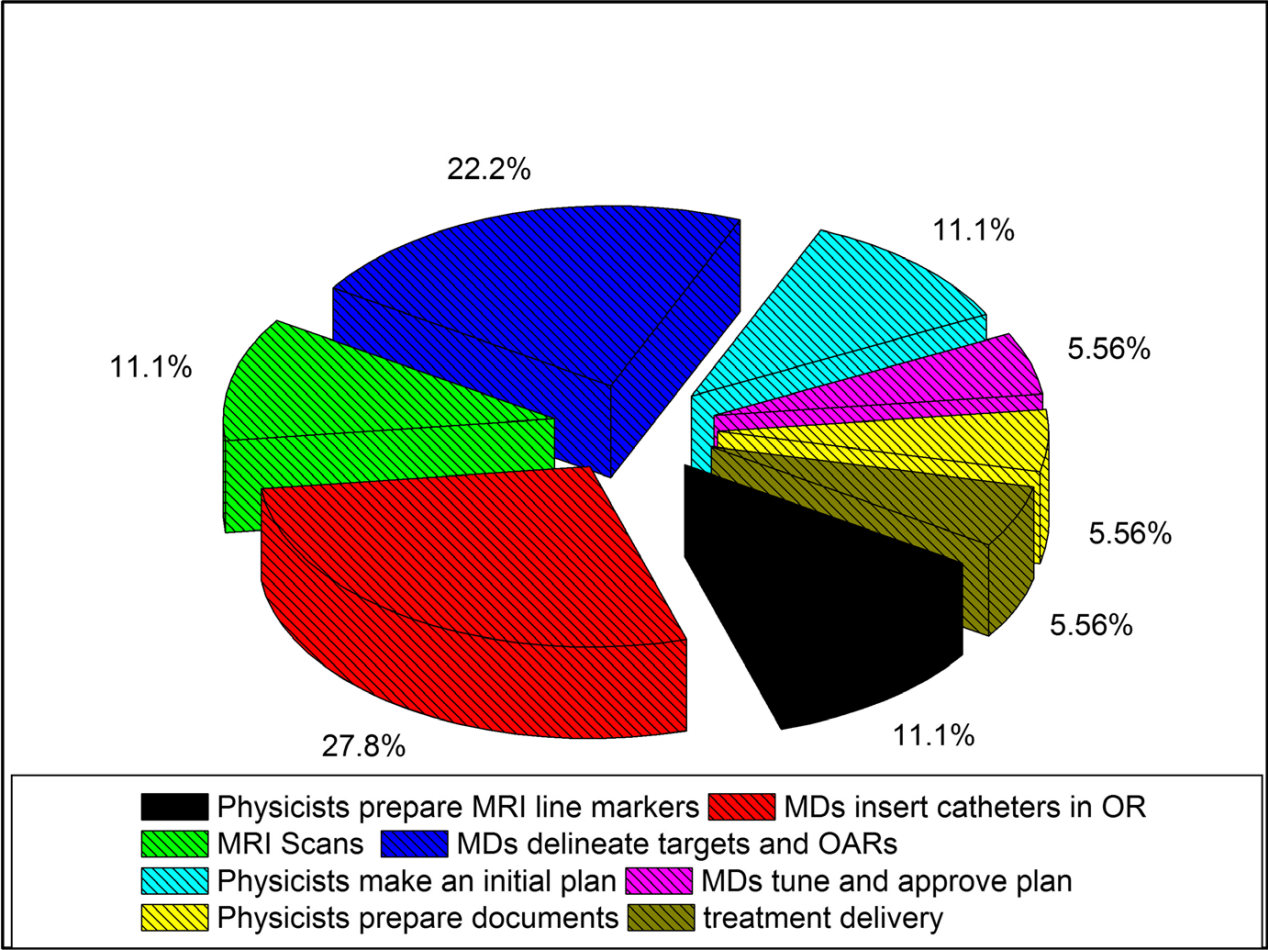
Pie map of the time budget for the procedure.

Although complicated, the entire procedure takes approximately 9 h in total and can be divided into 8 major parts. The Table 3 presents the average time of each part. The first work is the MRI line marker preparation which happened one day before the treatment day. Other sub‐procedures happened on the treatment day. In brachytherapy planning room two hours of time is budgeted for MD’s target delineation, this includes teaching residents, verifying resident’s work and tuning the initial treatment plan by attending MD. If teaching is not involved, the target delineation could be done in one hour in our institution. Figure 9 is the pie map of the time budget for completing the procedure. It indicated that the insertion of catheters in OR and delineation of targets and OARs in brachytherapy planning room by MDs account for half of the procedure time (Fig. 9).

**Table 3:** The times needed for each sub‐parts of the procedure for delivering first fraction of treatment.

Sub‐parts of the procedure	Time (hours)	Location
MRI line marker preparation	1	Physics room
Catheter insertion	2.5	Operation room
MRI scans	1	MRI lab of radiology dept.
MD contouring targets and OARs	2	Brachytherapy planning room
Physicist making an initial plan	1	Brachytherapy planning room
MD tuning/approving plan	0.5	Brachytherapy planning room
Physicists preparing documents, performing second check	0.5	Brachytherapy planning room
Treatment delivery	0.5	Brachytherapy suite

Abbreviation: MRI, magnetic resonance imaging.

## 5 CONCLUSION

MRI guided interstitial brachytherapy is achievable in most radiation oncology clinics if the required equipment is available. The equipment includes MRI scanner, CT scanner, HDR afterloader, CT/MRI‐compatible templates and applicators, MR line markers and a brachytherapy TPS capable of utilizing MRI images. The OR/procedure room availability and anesthesiology support are also very important. Careful physics equipment preparation and planning will facilitate the implementation of the procedure, and thus improve the treatment experience of the brachytherapy team and importantly the patients.

## CONFLICT OF INTEREST

The work was not supported, funded, or sponsored by any extra‐institutional source, nor are there any actual or potential conflicts of interest with the production or publication of this work. No author has any direct or indirect commercial financial incentive associated with publishing this article.
